# Pyroclastic Dust from Arequipa-Peru Decorated with Iron Oxide Nanoparticles and Their Ecotoxicological Properties in Water Flea *D. magna*

**DOI:** 10.3390/nano14090785

**Published:** 2024-04-30

**Authors:** Juan A. Ramos-Guivar, Yacu V. Alca-Ramos, Erich V. Manrique-Castillo, F. Mendoza-Villa, Noemi-Raquel Checca-Huaman, Renzo Rueda-Vellasmin, Edson C. Passamani

**Affiliations:** 1 Grupo de Investigación de Nanotecnología Aplicada para Biorremediación Ambiental, Energía, Biomedicina y Agricultura (NANOTECH), Facultad de Ciencias Físicas, Universidad Nacional Mayor de San Marcos, Av. Venezuela Cdra 34 S/N, Ciudad Universitaria, Lima 15081, Peru; 12130002@unmsm.edu.pe (Y.V.A.-R.); emanriquec@unmsm.edu.pe (E.V.M.-C.); freddy.mendoza1@unmsm.edu.pe (F.M.-V.); renzo.vellasmin@edu.ufes.br (R.R.-V.); 2 Centro Brasileiro de Pesquisas Físicas (CBPF), R. Xavier Sigaud, 150, Urca, Rio de Janeiro 22290-180, Brazil; nomifsc@cbpf.br; 3 Physics Department, Federal University of Espírito Santo, Vitória 29075-910, Brazil; passamaniec@yahoo.com.br

**Keywords:** hybrid materials, magnetic composites, secure materials development, composite ecotoxicity, pyroclastic dust materials, Peruvian Sillar

## Abstract

A novel magnetic composite made of Peruvian pyroclastic dust material decorated with maghemite nanoparticles was synthesized and characterized using a variety of analytic techniques. The 13 nm maghemite nanoparticles were grown on the pyroclastic dust using the conventional coprecipitation chemical route. A short-term acute assay was developed to study the ecotoxicological behavior of the water flea, *Daphnia magna*. A 24 h-lethal concentration (*LC*_50_) value equal to 123.6 mg L^−1^ was determined only for the magnetic composite. While the pyroclastic dust material did not exhibit a lethal concentration, it caused morphologically significant changes (*p* < 0.05) for heart and tail parameters at high concentrations. Morphologies exposed to the magnetic composite above the 24 h-*LC*_50_ revealed less tolerance and significant changes in the body, heart, antenna, and eye. Hence, it affects biomarker growth and swimming. The reproduction rate was not affected by the raw pyroclastic dust material. However, the number of individuals showed a decrease with increasing composite concentrations. The present study indicates the *LC*_50_ value, which can be used as a reference concentration for in-situ water cleaning with this material without damaging or changing the *Daphnia magna* ecosystem.

## 1. Introduction

Sillar is a pyroclastic dust material that is found near volcanic rock deposits. In the Arequipa region of Peru, this raw material is used for a house construction technique named as ashlar. Sillar has unique physical and mechanical properties, such as porosity and compressive strength, which make it an ideal material for construction [1]. Due to its nature, it is also known as ignimbrite, since it is the product of pyroclastic flows of explosions that occurred on the earth’s surface, from which a large number of magmatic substances similar to foams were released. These substances were crystallized under the influence of pressure and temperature, forming certain crystallographic phases (albite, biotite, cristobalite, plagioclase, and sanidine, among others) embedded in a matrix of broken glass residues [1,2,3,4]. In fact, there are some differences between ignimbrites and Sillar, i.e., ignimbrites have a combined glass/crystalline texture due to the expansion of volcanic gases during eruption, while Sillar has a more pronounced crystalline texture due to the presence of cristobalite, anorthoclase, and albite.

Sillar possesses interesting mechanical properties, e.g., its compressive strength, being optimal and above the recommended Peruvian Guidelines [3]. More important, it has been shown that Sillar is an optimal precursor material to produce zeolitic materials with remarkable adsorbent properties for various heavy metal ions such as Pb (II), Cd (II), and Cr (VI) [2,5,6].

In addition, to improve Sillar properties and broaden its technological applications, surface modification with nanoparticles (NPs) has recently emerged as a promising strategy. For example, Sillar composite samples have shown excellent physical and mechanical properties [1,4]. On the other hand, among the different NPs, iron-oxide nanoparticles (IONPs) would be a good choice because they have attracted considerable attention from the scientific community due to their unique magnetic and physicochemical properties and their biocompatibility [7]. However, Sillar modified with IONPs has not yet been studied or reported in the literature. Therefore, Sillar as a stabilizer (substrate) of NPs is still in the first stage of several studies [3,4], suggesting that it needs more studies and tests, not only for new technological applications, but also for checking their ecotoxicological effects. In addition, considering that these composite samples could be used in various fields(fast environmental remediation of contaminated systems, like aqueous effluents), they need to be evaluated not only for remediation applications but firstly prioritizing their ecotoxicity. This means that the determined lethal concentrations for these composites can be used to establish permissible limits for clean water effluents without damaging or causing changes in the ecosystem.

The ecotoxicity of novel adsorbent materials applied to biomarkers, such as *Daphnia magna* (*D. magna*), is currently under development [8]. This species was chosen because *D. magna* is often used as a biological indicator, i.e., it unequivocally reveals the adverse effects of chemicals in the aquatic environment. Exposure to specific compounds can provoke negative responses in this organism, affecting its reproduction and survival, giving us an indication of the impact of the adsorbent in the aquatic medium [9].

In this work, the first study on the synthesis, characterization, and ecotoxicological evaluation of Sillar decorated with IONPs was conducted. The purpose of this study was to evaluate the toxicity of both materials in *D. magna*. For that, a short-term acute assay for 24 of exposure time was developed. Therefore, we found the lethal concentration correlated with the adsorbent dose often used in adsorption experiments for water treatment at the laboratory scale. Finally, this study will reinforce the current state of knowledge regarding the toxic effects of the studied materials on *D. magna*, its morphological changes, and the number of individuals exposed to various concentrations.

## 2. Materials and Methods

### 2.1. Adsorbent Synthesis

For the synthesis of IONPs, the following chemical reactives were used: ferrous sulfate heptahydrate (FeSO_4_•7H_2_O), iron (II) chloride (FeCl_3_), ammonium hydroxide (NH_4_OH) at 28% *v*/*v*, all of them from Sigma Aldrich.

### 2.2. Synthesis of IONPs by Coprecipitation Method

The IONPs were synthesized using the coprecipitation method [10]. In a first step, 100 mL of ultrapure water was heated to a constant temperature of 80 °C and left stirring at 400 rpm. Second, iron salts in a molar ratio of Fe^+2^/Fe^+3^ = 0.5 were dispersed in the heated water reservoir with subsequent addition of 15 mL of NH_4_OH and the reaction was allowed to proceed while stirring for 30 min, keeping the same temperature. Third, the resultant material was washed several times by magnetic decantation assistance, until a neutral pH was reached. Finally, the recovered solid sample was dried at 80 °C for 24 h for subsequent grinding with a porcelain mortar.

### 2.3. Sillar from Añhashuayco Quarry

**Figure 1:**
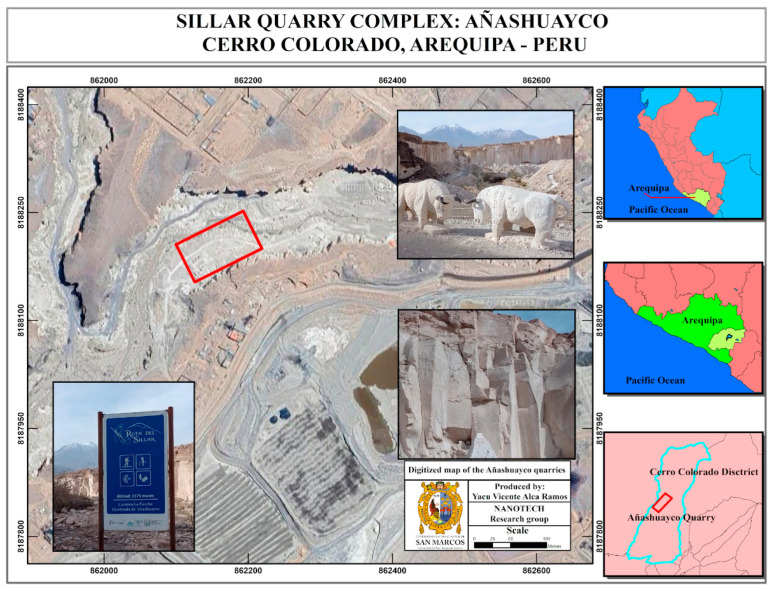
Thematic map of the Añashuayco quarry, located in Arequipa. The red box corresponds to the sector where the Sillar was extracted for the present work whose name is “La Paccha”.

The Añashuayco Creek, born at the confluence of three streams near the Añashuayco Bridge (16°21′48.5” S 71°36′31.5” W), within the so-called “Rutas del Sillar”, is located within the district of Cerro Colorado. The samples were collected directly from the Añashuayco quarries in the sector known as La Paccha (Figure 1). Initially, medium-sized rocks were extracted, which for the analysis were subjected to mechanical crushing to reduce them to dust and thus, be able to perform the corresponding measurements for their characterization.

### 2.4. Synthesis of the Composite

For the synthesis of Sillar decorated with IONPs, 9.907 g of Sillar were dispersed in 226 mL of distilled water under speed agitation of 550 rpm for 30 min until the dispersion was stable. Thereafter, 6.5 g of γ-${Fe}_{2}O_{3}$ NPs, previously synthesized by the chemical coprecipitation method, were added and the resulting dispersion was left stirring at 250 rpm for 24 h. Then, the material was washed and dried in an oven at 65 °C for 24 h. Finally, it was ground with a mortar, obtaining 16.5 g of dark brown compound. This composite was labeled as the MS sample, and this nomenclature was used ahead.

### 2.5. X-ray Diffraction Measurements

To determine the crystalline structure and crystallite size by X-ray diffraction (XRD) the Rigaku equipment, Ultima IV, in Bragg-Brentano configuration was used. All the diffracted intensities were recorded employing CuK_α_ radiation and at 40 kV and 30 mA current in a scanning range from 20° to 80°. The instrumental resolution function for instrumental broadening correction was obtained from standard CeO_2_ material (see the supplementary material section). Crystallographic identification was done using Match v3 software [11]. The crystallographic information files 900-8111 (cristobalite; SiO_2_), 901-3309 (anorthoclase; Al_1.1_Ca_0.1_K_0.27_Na_0.63_O_8_Si_2.9_), 900-9663 (sanidine; AlKO_8_Si_3_), and 900-0526 (albite; AlNaO_8_Si_3_) were used to identify the corresponding phases, and OriginPro 9.0 software was used to determine the full width at half maximum (β) by means of a Gaussian approximation for each peak corresponding to the different identified phases.

To get information about mean crystallite sizes of these phases, the Scherrer integral breadth method following Equation (1) was used:

(1) $$D=\frac{K\lambda }{\beta_{D}cos\theta }$$

where *D* is the crystallite size (nm), *K* is a shape constant, λ is the wavelength of the incident X-rays (Å), $\beta_{D}$ is the corrected peak broadening, which depends on two factors as shown in Equation (2), and *θ* is the diffraction angle.

(2) $$\beta_{D}={\left[{\beta_{measurement}}^{2}-{\beta_{instrumental}}^{2}\right]}^{1/2}$$

### 2.6. Transmission Electron Microscopy Measurements

The equipment used to obtain transmission electron microscopy (TEM) images, where mean particle size, size distribution, and morphology were determined, was a 200 kV JEOL 2100FX microscope (Tokyo, Japan). The imaging microscopy instrument was used in transmission mode and with high resolution. For the estimation of Particle Size Distribution (PSD), a previously reported methodology was used [12]. To obtain robust statistical data, 800 to 1000 particles were counted from 20 to 25 images using ImageJ software v 154. For pure NPs, 800 particles were counted, and for the MS sample, 1350 particles were counted. Subsequently, histograms were fitted using a log-normal distribution. The polydispersity index (PDI) values were determined using the ratio between the standard deviation and the mean particle size of the log-normal distribution.

### 2.7. Thermogravimetric Measurements

For thermogravimetric analysis (TGA), the Shimadzu TGA-50 Series thermogravimetric instrument (Shimadzu Corporation, Kyoto, Japan) was used in an oxidizing air environment with a flow rate of 50 mL/min, a speed of 10 °C/min, and in a temperature range of 20 to 900 °C.

### 2.8. ^57^Fe Mössbauer Spectrometry

^57^Fe Mössbauer spectrometry was used to analyze the hyperfine magnetic structure of the novel composite [13]. Transmission ^57^Fe Mössbauer spectra were performed at 300 K and 15 K using an He closed cycle setup from Janis Corporation Inc. The Mössbauer spectrometer, operating with triangular or sinusoidal velocities, respectively, was mounted in-phase with the sample-holder, i.e., both installed in an antivibration system and strongly connected. A radioactive source of ^57^Co immersed in Rh matrix with 25 mCi was used. For the low spectra, the source was kept at room temperature (RT), while the absorber could be reduced to low temperatures. The powder absorbers were enclosed in nylon sample holders; their effective thicknesses were selected to be equivalent to 0.1 mg ^57^Fe per cm^2^. ^57^Fe Mössbauer spectra were fitted using Mosswinn 4.0i software [14].

### 2.9. Vibrating-Sample Magnetometry Measurements

For the Direct Current Vibrating-sample magnetometry (VSM) measurements, the Physical Properties Measurement System (PPMS) Evercool-II, from Quantum Design Inc.—San Diego, CA, USA, was used, with a Vibrating Sample Magnetometer module operating for a maximum field of ±6 T.

The values obtained for the saturation magnetization (*M*_s_) were obtained by adjusting the Law of Approach to Saturation (LAS) following Equation (3) [15],

(3) $$M=M_{s}\left(1-\frac{b}{H^{2}}\right)+\chi H$$

where *b* (kOe)^2^ is related to the effective anisotropy constant and *χ* (emu g^−1^ kOe^−1^) is magnetic susceptibility at high magnetic fields.

### 2.10. Dynamic Light Scattering and Zeta Potential

The Dynamic Light Scattering (DLS) and Zeta potential measurements were done using a Brookhaven Nanobrook 90 Plus PALS (Brookhaven Instruments Corporation, Holbrook, NY, USA).

### 2.11. D. magna Culture and Exposure Protocol

The effect of chemicals on the reproductive output of *D. magna* is often evaluated with the OECD TG 21 test [16]. However, this test is performed for young or adult individuals only and not female *D. magna* neonates. The purpose of using this composite adsorbent is to quickly eliminate contaminants in a water-based environment within a period of no more than 24 h. The short-term acute assay was optimized and standardized in the lab for this aim, as also reported in earlier publications [17,18].

For that, the culture of *D. magna* was carried out from a first maternal litter with the objective of reproducing them up to the third generation and then, subjecting them to ecotoxicity tests with the corresponding neonates. The conditions of this culture were a temperature of 20 °C, a pH of 7.5, and a photoperiod of 8(16) h of light(dark). These *D. magna* individuals were divided into four beakers (10 individuals per beaker) and placed in 200 mL of standardized water for *D. magna* culture. The corresponding neonates (female clones) were born after one to five days. Daily feedings of the biomarker consisted of 2 mL of *Chlorella vulgaris* microalgae. For the exposure experiments, only clone female individuals reproducing by parthenogenesis [19] were used—since male individuals exhibit genetic variability due to haploid eggs and different morphologies are expected, such as body and antenna sizes—that make possible their comparison [9,20]. In addition, exoskeleton molts were removed daily, and individuals were transferred to new beakers periodically. This process was continued until third-generation hatchlings were obtained under the optimal standardized conditions established in the laboratory.

The ecotoxicity experiments for different concentrations were done separately. In a volume of 200 mL of standardized water, four concentrations of Sillar sample were prepared: 500 mg L^−1^, 1000 mg L^−1^, 5000 mg L^−1^, and 10,000 mg L^−1^. While for the MS sample, six chosen concentrations were also prepared: 25 mg L^−1^, 50 mg L^−1^, 100 mg L^−1^, 150 mg L^−1^, 400 mg L^−1^, and 800 mg L^−1^. The number of neonates used for the ecotoxicity experiment was 10, as had been done in previous research [18]. Three replicates were done for each experiment.

For the dispersion of Sillar or MS composite, sonication was used for 15 min, and the total duration of the exposure experiment of Sillar or MS composite was 24 h. In addition, during the 24 h-ecotoxicity experiment, the biomarkers were not fed.

To determine if there were significant changes after 14 days of exposure to Sillar or MS composite, a morphological analysis was performed with respect to a negative control (N.C.) [18,21]. The measurements of the morphological parameters were performed using a Greetmed optical microscope, model DN117M (Leica Microsystems, Wetzlar, Germany), for each of the applied concentrations with a resolution of ×4 magnification. It is important to note that the concentrations applied were selected after an exhaustive review of the literature, i.e., concentrations that would not severely impact the living being species.

### 2.12. Data Analysis

To verify significance with the *D. magna* set, a N.C. was used to carry out the corresponding comparison. This analysis was performed using the Student’s t-test and Wilcoxon’s t-test with 95% confidence intervals. Prior to this, it was evaluated whether the data for the morphological parameters to be measured came from a normal distribution using the Shapiro-Wilk normality test. The determination of significance compared to the N.C. was based on a *p*-value equal to 0.05 [22]. Mortalities were expressed as Probit units. Linear regression was carried out using OriginPro 9.0 software. The goodness of the linear fit was proven using the metric R-squared, R^2^.

## 3. Results and Discussion

### 3.1. XRD Analysis

**Figure 2:**
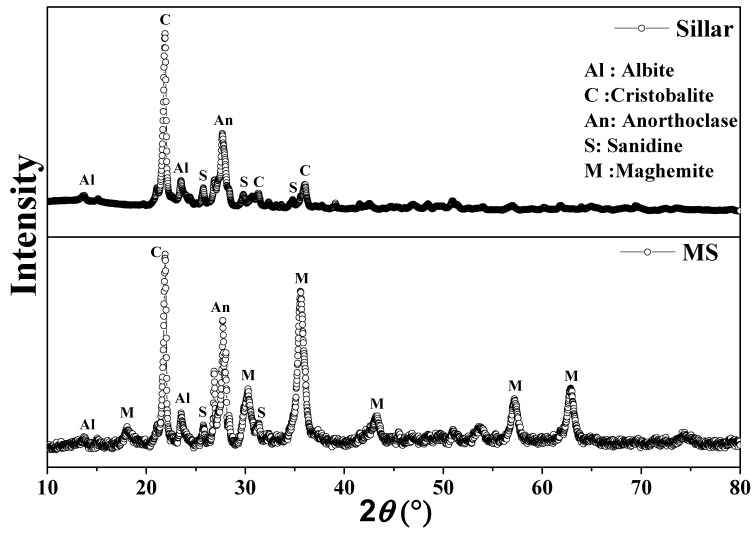
X-ray diffractograms of the Sillar and MS samples. The upper letters indicate the matching crystallographic phases; it can be observed the overlapping of the magnetic phase and Sillar forming the resulting magnetic composite.

Figure 2 shows the X-ray diffractograms for Sillar (top panel) and MS composite (bottom panel). The XRD analysis of the Sillar sample showed the presence of several mineralogical phases. The analysis identified Cristobalite (27 *wt* %), Anorthoclase (26.4 *wt* %), Sanidine (26.3 *wt* %), and Albite (20.3 *wt* %).

Tables S1 and S2 outline the parameters obtained from the Scherrer analysis, including *D* values for each crystalline phase.

Additional crystallographic information is provided for the pristine Sillar sample due to its abundance and extensive use in Peru. This leads to an in-depth explanation of the crystalline phases seen in Figure 2. First, the crystalline form of silicon dioxide (SiO_2_), known as cristobalite, occurs at high temperatures and has an atomically ordered three-dimensional structure [23].

On the other hand, Anorthoclase and Albite are two minerals belonging to the feldspar group [24]. Anorthoclase is a plagioclase feldspar of variable composition, while Albite is a plagioclase feldspar rich in sodium. The presence of these phases suggests that the pristine sample of the Arequipa city may be igneous or metamorphic rock, as feldspars and Cristobalite are common in these rocks. The mineralogical composition indicates that there is a relatively high proportion of Albite feldspar in the sample, followed by a similar amount of Anorthoclase and Cristobalite. As mentioned in the introduction, an ignimbrite is a volcanic rock of pyroclastic origin that forms during explosive eruptions. It is composed mainly of volcanic glass fragments and mineral crystals and is characterized by a vesicular texture and a glass matrix [25]. Thus, the main difference between Sillar and ignimbrite is that while Sillar contains Cristobalite, Anorthoclase, and Albite in high concentration, indicating a magmatic or metamorphic origin, an ignimbrite is basically of volcanic origin formed in explosive eruptions when volcanic ash and pyroclastic fragments are ejected in a gaseous mixture and rapidly deposited to form a layer of solidified volcanic material. Ignimbrites may also contain minerals such as feldspar, quartz, and volcanic glass, while in Sillar, they are found in low fractions. The exact proportion and composition of these minerals may vary between both samples. Thus, the XRD results presented in Figure 2 suggest that the high concentration of Cristobalite, Anorthoclase, and Albite indicates a composition different from that expected for an ordinary ignimbrite.

On the other hand, in the Sillar decorated with IONPs, the phases were determined with the contribution of γ-Fe_2_O_3_ in the following percentages: Sanidine (29 *wt* %), Cristobalite (26 *wt* %), Albite (19.8 *wt* %), γ-Fe_2_O_3_ (24.1 *wt* %), and Anorthoclase (1.1 *wt* %). When Sillar is modified (MS sample) new properties arise, for example, one enhances the magnetic properties of the composite. Hence, this new property allows the control and manipulation of the composite by means of external magnetic fields, which have applications in environmental remediation [26,27]. It is important to keep in mind that the effects of Sillar surface modification with γ-Fe_2_O_3_ will depend on several factors, such as the amount of iron-oxide used, the procedure in which it is incorporated into the Sillar, the distribution of the γ-Fe_2_O_3_ NPs, and the chemical and magnetic interactions between the involved crystalline phases. In addition, detailed characterization, and study of the properties of the magnetically decorated Arequipa Sillar would be necessary to fully understand the effects of this modification on the material.

### 3.2. TGA Analysis

**Figure 3:**
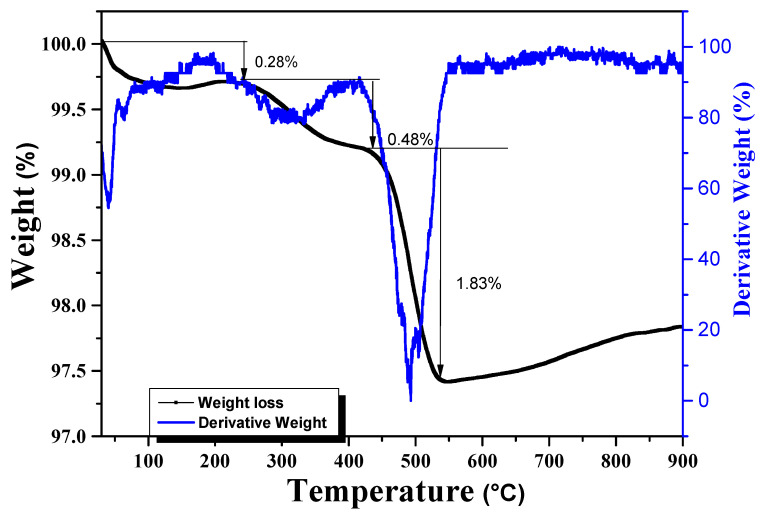
TGA and DTA graphs for the MS sample. Each value in percentage inserted in the figure is associated with the black line and represents the weight loss step of each process.

Plagioclasts are a group of minerals that belong to the feldspar series, which includes potassium feldspar and sodium and calcium feldspars. The TGA of plagioclasts can provide information about their thermal stability and related properties. One of the most important events that can be observed during thermogravimetric analysis of plagioclasts is moisture loss; plagioclasts may contain moisture adsorbed on their surfaces or even within their structure [28]. Figure 3 displays the thermogravimetric curve (and its derivative) obtained for the MS sample. It is possible to observe an initial weight loss due to the desorption of this moisture at relatively low temperatures (ca. 100 °C), and then dehydration occurs up to 450 °C. Some plagioclasts, such as calcic and/or potassic plagioclase, may contain traces of structurally bound water in their crystal lattice. Therefore, at higher temperatures (>300 °C), this structural water may experience dehydration, which is reflected as an additional mass loss up to ca. 425 °C, and finally, there is thermal decomposition at higher temperatures (>425 °C). These two last events may be related to the release of gases such as carbon dioxide, which may occur due to the decomposition of carbonates present in the sample or due to the restructuring of the feldspar under high temperature conditions. As a result, the aforementioned processes can manifest as changes in weight loss in a thermogravimetric analysis, as shown in Figure 3 [5]. It is important to note that the events observed during thermogravimetric analysis of plagioclasts may vary depending on the exact composition of the plagioclast, as the compositional range extends from sodium feldspars to calcium feldspars.

More specifically, the thermogram for the MS sample showed that at 100 °C, there is no elimination of volatile compounds. At a temperature of 221 °C, there is the first point of elimination of volatile products with a weight loss of 0.28%, which could correspond to small traces of water structurally bound in its crystalline network found on the surface. Increasing the sample temperature, a weight loss of 0.48% occurred at 403 °C, likely related to the transformation of γ-Fe_2_O_3_ to hematite. Finally, a weight loss of 1.83% occurred at 532 °C. On the other hand, in the region of 550 to 900 °C, a weight gain can be regarded. This is presumably due to the entrapment of air molecules that remained in the Sillar pores since it is a clay material. In fact, this behavior is typically found in micro/mesoporous-like cavities and some types of aluminosilicate compounds, such as zeolites [29,30].

### 3.3. TEM Analysis

**Figure 4:**
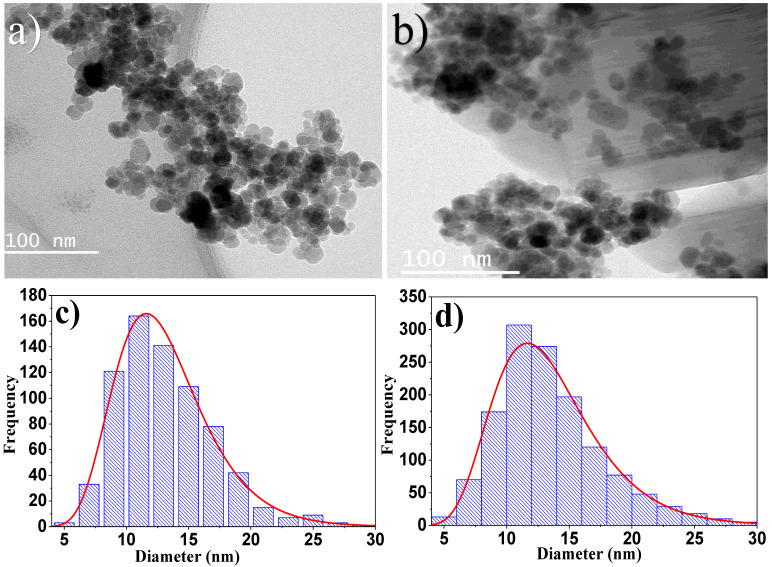
TEM images of (**a**) pure γ-Fe_2_O_3_ NPs and (**b**) MS samples. In (**b**), the pure γ-Fe_2_O_3_ NPs (2D black-like morphologies) are dispersed into the Sillar sample (big supporting matrix in grey color). PSD for (**c**) pure γ-Fe_2_O_3_ NPs and (**d**) MS sample. The red line indicates the best fit using a log-normal distribution.

TEM images of the as-prepared γ-Fe_2_O_3_ NPs and MS composite are, respectively, shown in Figure 4a,b. The TEM image shown in Figure 4b suggests that Sillar has acted as a layer that harbors the γ-Fe_2_O_3_ NPs in some regions of its surface, i.e., it serves as a substrate for the dispersion of the γ-Fe_2_O_3_ NPs. The PSD for both materials is given in Figure 4c,d. The PSD histogram fit gave a mean particle size value of the γ-Fe_2_O_3_ NPs that decorate Sillar of 12.7 (1) nm. This was a value similar to that found in as-prepared γ-Fe_2_O_3_ NPs, 12.5 (3) nm. Therefore, the TEM result indicates that the decoration of Sillar with γ-Fe_2_O_3_ NPs has not generated a significant change in the γ-Fe_2_O_3_ size, so a strong chemical interaction between Sillar and γ-Fe_2_O_3_ NPs may be negligible. However, since the γ-Fe_2_O_3_ NPs decorate the Sillar surface, a small chemical affinity must be considered. Possible chemical interactions that should be assumed are, at least, electrostatic interactions or weak chemical bonds (like van der Waals interactions) that favor adhesion and the formation of a cohesive interface. Another possibility would be the physical adsorption of the γ-Fe_2_O_3_ NPs where the morphology and porous structures of the Sillar could allow the incorporation and retention of the γ-Fe_2_O_3_ NPs in its matrix, but this assumption is not clear in Figure 4b. Thus, the texture of the Sillar could provide anchoring sites and a surface conducive to adsorption and NP deposition. Moreover, the presence of the Sillar, as a substrate, contributes to the stabilization and dispersion of the γ-Fe_2_O_3_ NPs, preventing an enhancement of agglomeration and aggregation of γ-Fe_2_O_3_ NPs.

To conclude this section, it can be noted that there are no reported works where the effect of the Sillar on magnetic NPs can be observed, like for magnetic NPs in zeolites, e.g., therefore it is hard to compare the polydispersity effect since it will depend on several factors, such as chemical affinities of the constituents and relative fractions, among others. On the other hand, the PSD fit indicates that this compound tends to maintain uniform poly-dispersion, with the PDI value for the MS sample equal to 0.01, which agrees with the single γ-Fe_2_O_3_ NP reporting a PDI value of 0.02. It is smaller than the previously reported (0.17–0.3) value for core-single-shell and core-double-shell polymer functionalized magnetic NPs [12]. Therefore, the PSD for γ-Fe_2_O_3_ NPs is not affected when decorating Sillar surface.

### 3.4. VSM Analysis

**Figure 5:**
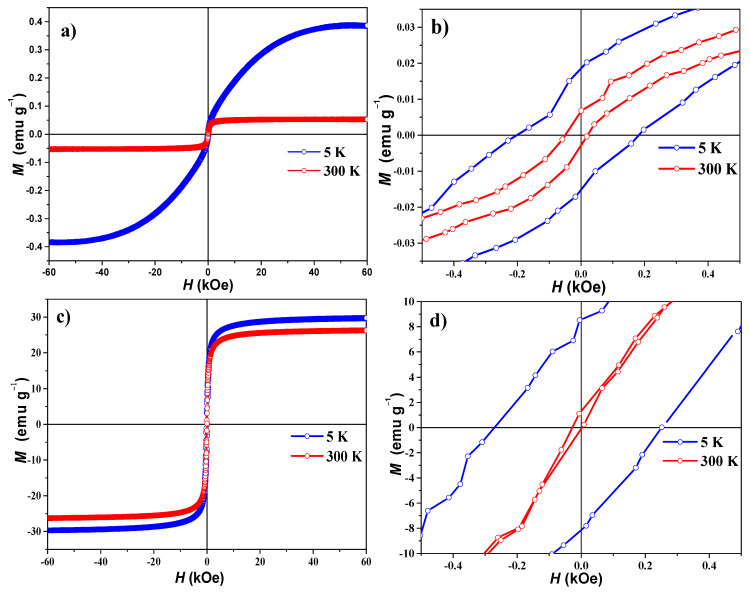
(**a**) M(H) curve for the Sillar sample at 5 K (blue dots) and 300 K (red dots), (**b**) their respective magnification at low magnetic field amplitude, (**c**) M(H) curve for the MS sample at 5 K (blue dots) and 300 K (red dots), (**d**) their respective magnification at low magnetic field amplitude.

The magnetic measurements were taken at two temperatures (300 K and 5 K) for both the pristine Sillar sample and the MS composite and the data are displayed in Figure 5. From the magnetic point of view, it can be said that Sillar has a low coercive (*H*_c_) field and small value of magnetization per gram compared to the MS sample. So, the Sillar sample is easily demagnetized, but its ferromagnetic-like character shown in Figure 5a,b suggests a presence of magnetic contribution that could be due to a weak magnetic structure and/or a presence of a small fraction of magnetic Fe-species, as those found in ^57^Fe Mössbauer spectroscopy [31].

More specifically, the Sillar sample has a coercive field (*H*_c_) of 0.035 kOe, remnant magnetization (*M*_r_) of 0.005 emu g^−1^, and a *M*_s_ value of 0.053 emu g^−1^, the latter suggesting that the sample has a moderate magnetization capacity in its pristine state at 300 K, as mentioned above. The *M*_S_ value depends on the composition and magnetic structure of the material, as well as the intrinsic magnetic properties of the components present in the ignimbritic Sillar. On the other hand, the magnetic measurement made at 5 K for the Sillar sample shows a *H*_c_ field of 0.2 kOe, *M*_r_ of 0.02 emu g^−1^, and a *M*_s_ of 0.43 emu g^−1^, indicating that this material has a greater magnetization capacity at lower temperatures, as would be expected by reducing the thermal effect and favoring the magnetic interactions responsible for the magnetization of the sample.

LAS analysis of the MS sample showed a better magnetic response (Figure 5c,d), since at 300 K, a reduction of *H*_c_ was observed with a value of 0.015 kOe and an increase in *M*_s_ of 26 emu g^−1^, while at 5 K, *H*_c_ showed a slight increase to 0.26 kOe and its *M*_s_ increased to 29 emu g^−1^. Additional fit values for the Sillar sample were b = 6.72 (kOe)^2^, χ = −1.24 × 10^−5^ (emu g^−1^ kOe^−1^) and b = 156.51 (kOe)^2^, χ = −4.57 × 10^−4^ (emu g^−1^ kOe^−1^) at 300 K and 5 K, respectively. For the MS sample, b = 4.42 (kOe)^2^, χ = 0.012 (emu g^−1^ kOe^−1^) and b = 5.35 (kOe)^2^, χ = 0.017 (emu g^−1^ kOe^−1^) for 300 K and 5 K, respectively. In addition, the *M*_r_ value for MS sample was found to be 8.35 emu g^−1^ at 5 K. The S-shape of the *M*(*H*) curve of both the Sillar sample (Figure 5a,b) as well as the MS composite sample (Figure 5c,d), shows the influence of temperature as well as the effect of the NPs on the magnetic response for S. As described in Section 2, the contribution of the NPs is visible in the *M*(*H*) curves due to the γ-Fe_2_O_3_ phase identified in the sample when the XRD was performed, since the contribution of this phase in the sample is (0.24:1) with respect to the other phases. And according to [32], it is mentioned that the percentage in mass of the magnetic part, in some cases, shields the Bragg diffracted peaks, which are the most outstanding peaks of the magnetic phase in the diffractogram. It should also be noted that being a composite of several phases, mostly tectosilicates, the magnetic contribution is very little, with respect to the iron NPs as in the case of γ-Fe_2_O_3_ and this is reflected in the magnetic response of the MS sample. Finally, the Stoner Wohlfarth ratio (*M*_r_/*M*_s_) was determined from the 5 K *M*(*H*) curve, to have values of 0.29 and 0.05 for the MS and Sillar samples, respectively, while the theoretical value is 0.5, which indicates that they exhibit an interacting behavior with the multidomain magnetic structure [12].

### 3.5. ^57^Fe Mössbauer Spectrometry Analysis

**Figure 6:**
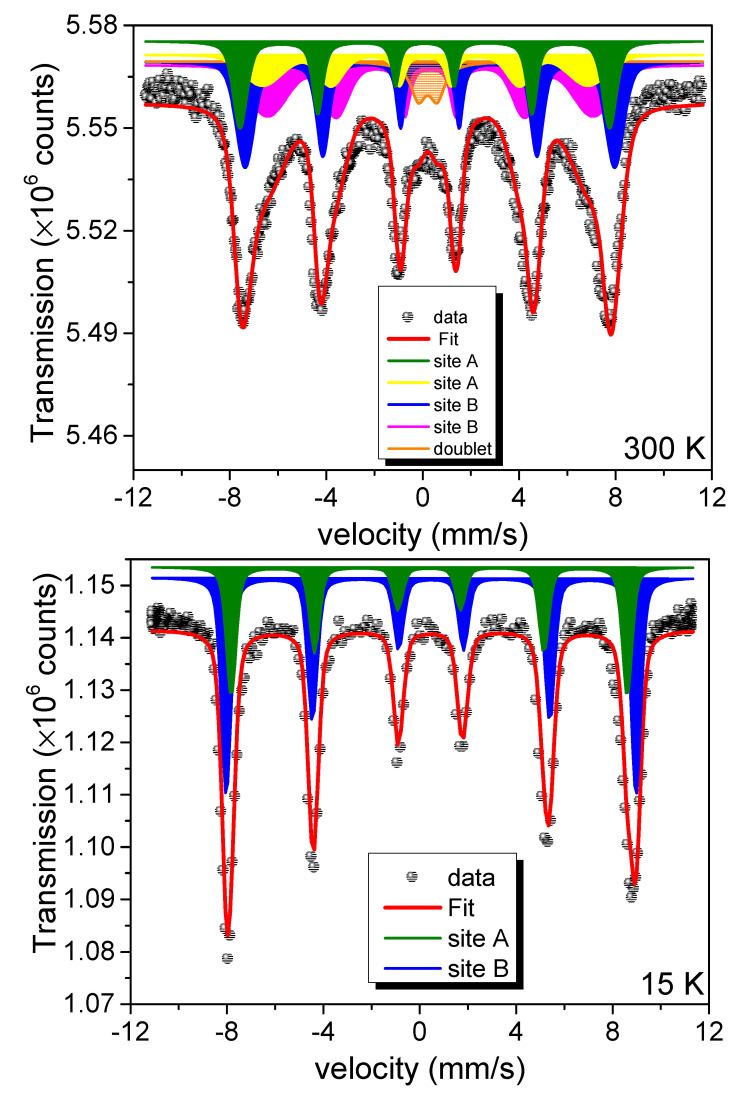
^57^Mössbauer spectra for the MS sample recorded at 300 K and 15 K. The subspectra used to fit the spectra are discussed in the text and shown with different colors in the figure.

To elucidate the magnetic behavior of the MS sample with respect to the inverse spinel magnetic phase, the ^57^Fe Mössbauer results obtained at low (15 K) and high temperature (300 K) are shown in Figure 6. Tables S3 and S4 show the hyperfine parameters obtained from the fits of the spectra at 300 and 15 K, respectively.

While at 15 K, two subspectra well fit the Mössbauer spectrum, at 300 K, the fit was performed with five components. The first two components were assigned to two static sextets corresponding to the bulk tetrahedral (A) and octahedral (B) sites, both related to the Fe^+3^ spins for the inverse spinel structure of the γ-Fe_2_O_3_. The third and fourth component were related to the dynamic magnetic behavior of trivalent iron spins characterized by two relaxing patterns. Theoretically, they are described by the Blume–Tjon two-level relaxation model [33] describing magnetic interacting systems, as observed by TEM images. In addition, the fifth component corresponds to a doublet, belonging to smaller particles at the superparamagnetic regime.

At 15 K, as mentioned above, the spectrum was fitted with the A and B sites of iron spins. The results of this fit are shown in Table S4. The expected ratio between the relative absorption areas for both sites was 3/5 as expected for the γ-Fe_2_O_3_ phase [21]. The values for the isomer shift of the two Fe sites agree that non-divalent spin states were formed during magnetic composite synthesis, so our γ-Fe_2_O_3_ NPs are atomically ordered even with sizes ca. 13 nm found in TEM results. From the TEM and Mössbauer results, it can be inferred that no new Fe-phase, that could be formed after chemical interaction between γ-Fe_2_O_3_ and Sillar, was observed by ^57^Fe Mössbauer spectroscopy and the subspectra used to fit the 300 K spectrum are commonly found in γ-Fe_2_O_3_ NPs (sizes larger than 10 nm) prepared by the coprecipitation method that often show a broad PSD [31].

### 3.6. Colloidal Stability Analysis

**Figure 7:**
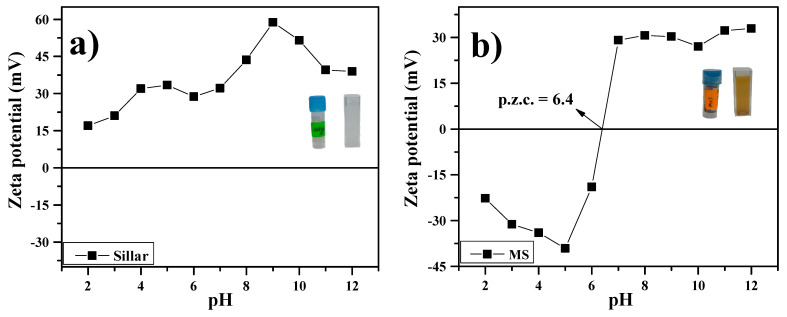
Zeta potential vs. pH graphs for (**a**) pure Sillar and (**b**) MS samples at neutral pH, which is ideal for application in water systems. The insets in the subfigures represent the ideal color for the aqueous suspensions ready for measurements. p.z.c. indicates the point of zero charge for the magnetic composite (MS).

The hydrodynamic diameters determined for the suspensions (pH = 7) of each sample by the DLS technique for the Sillar and MS samples were 609 and 379 nm, respectively, which contrasts with the assumption that the Sillar used acts as a cover on the γ-Fe_2_O_3_ NPs. On the other hand, the pH dependence of the zeta potential measurements was used to determine the point of zero charge (p.z.c.), these being those where the net charge is zero within a given pH range. For the MS sample, a p.z.c. was found at pH 6.4, observing negative values below this one, which show us, in some way, the contribution of the γ-Fe_2_O_3_ NPs within the Sillar, because in Figure 7a, it can be noted that for pH higher than 7, it has an action of the Sillar in comparison to Figure 7b, while for lower pH, it is a contribution of the γ-Fe_2_O_3_ NPs. For the Sillar sample, it was not possible to determine the p.z.c. because positive charges were shown in all the pH range, and this is probably due to the anhortoclase phase of this compound, since this phase presents the characteristic cations Ca, K, and Na. Furthermore, the p.z.c. allows us to assert that the magnetic composite does not require any chemical process to stabilize the pH in terms of its application in aqueous systems, which is convenient for environmental applications.

### 3.7. Acute Toxicity of Sillar and MS in D. magna

#### 3.7.1. Lethal Concentration 24 h-LC_50_

The ecotoxicity experiment was carried out with the purpose of determining the *LC*_50_ value in the Sillar and MS samples, with an exposure duration of 24 h. The results revealed that the Sillar sample did not present a discernible *LC*_50_. Even at high concentrations, such as 10,000 mg L^−1^, the mortality was practically the same as that observed at a concentration of 2500 mg L^−1^. Furthermore, it could be added that Sillar sample precipitation caused the neonate deaths at the highest concentrations.

**Figure 8:**
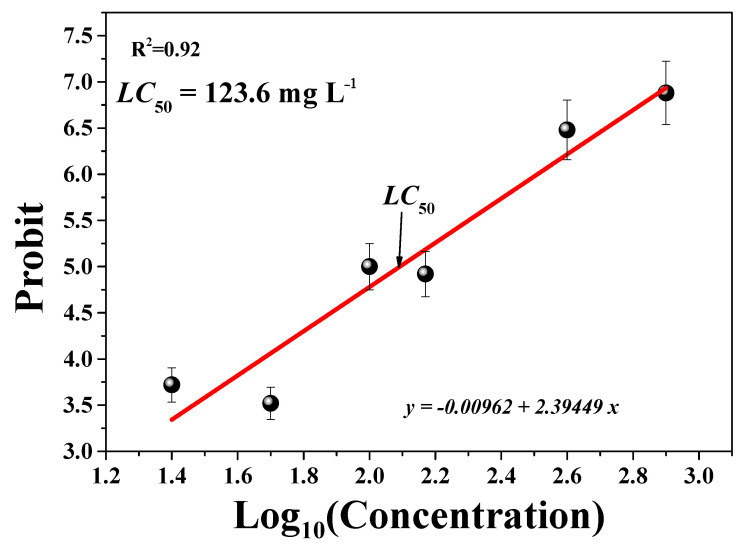
Graph corresponding to lethal concentration for the MS sample. Y-axis is presented in Probit numbers. The arrow indicates the coordinate at which the *LC*_50_ value was determined.

In contrast, the MS sample showed an *LC*_50_ of 123.6 mg L^−1^, whose linear equation is shown in Figure 8.

Where ***y*** is the Probit number of the corresponding mortality percentage and ***x*** represents the logarithm of the concentration, thus obtaining a linear fit of R^2^ equal to 0.92. In addition, the concentration at which 100% of the *D. magna* individuals died was 1200 mg L^−1^.

As mentioned above, in the literature, there are not many works that perform the ecotoxicological study of composites similar to this work, but there are references that make known nanohybrids based on functionalized γ-Fe_2_O_3_ NPs. For example, for the ternary nanocomposite made of graphene oxide functionalized with γ-Fe_2_O_3_ NPs and titanium dioxide, the *LC*_50_ was 550 mg L^−1^ [34], while for pure anatase, titanium dioxide, it was 166 mg L^−1^ [18]. The presented results are of utmost importance for the adsorbents development because the *LC*_50_ value agrees with the reported adsorbent doses proposed for water cleaning using adsorption processes at the laboratory scale [25,34,35].

These results suggest that the *LC*_50_ of the MS sample is in the intermediate range of the reported toxicity in the literature. More importantly, the *LC*_50_ determination is attributed to the presence of the γ-Fe_2_O_3_ NPs (24.1 *wt* %), likely indicating a size-effect dependence when contrasting with the bulk Sillar sample.

#### 3.7.2. Morphological Analysis in *D. magna*

**Figure 9:**
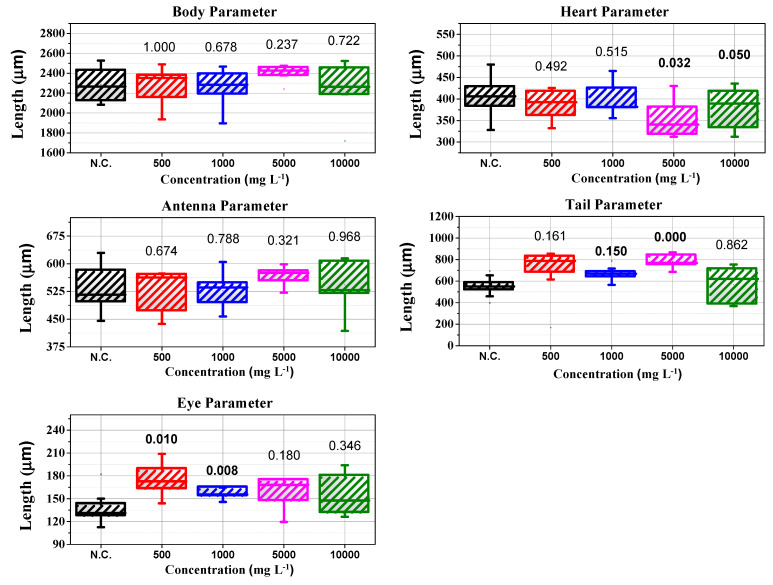
Morphological parameters of *D. magna* exposed to the Sillar sample, where the parameters that had significant changes were heart, antenna, and eye. Numbers above boxes are their *p*-values, with those written in bold numbers indicating significance (*p*-value < 0.05). N.C. stands for negative control.

**Figure 10:**
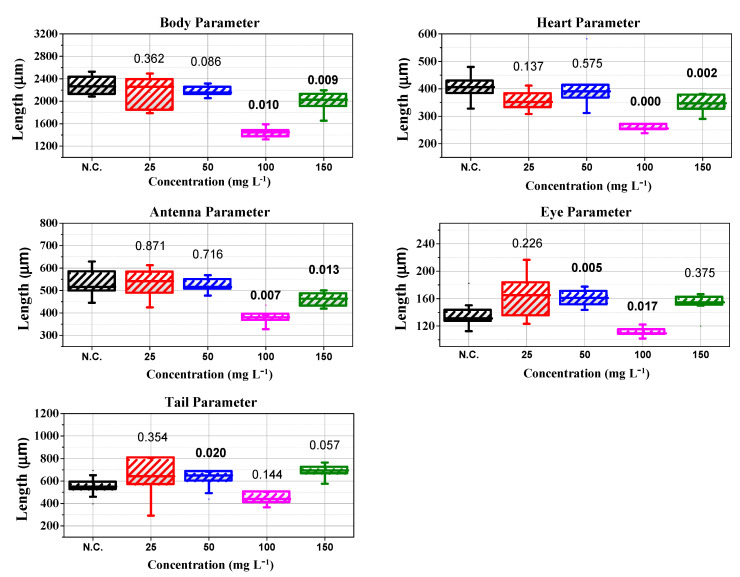
Morphological parameters of *D. magna* exposed to the MS sample, where the parameters that had significant changes were heart, antenna, and eye. Numbers above boxes are their *p*-values, with those written in bold numbers indicating significance (*p*-value < 0.05). N.C. stands for negative control.

Figure 9 and Figure 10 show the box plots corresponding to the morphological evaluations after 14 days of exposure to Sillar and MS samples. These graphs are important because they allow the determination of the *p*-value when comparing exposure lengths to N.C. data [22,36].

The results in Figure 9 revealed that, although there is not a *LC*_50_ value for the Sillar sample, the morphological parameter lengths varied. In addition, statistical significance for the heart and tail length parameters was observed for the high concentrations. While for the eye parameter, the statistical significance occurs for low Sillar concentrations. These findings indicate that the growth and swimming characteristics of the body and antenna parameters of *D. magna* are not affected when exposed to the various Sillar sample concentrations.

In contrast, in Figure 10, morphological changes were observed for all the parameters exposed to the MS sample. Particularly, the body, heart, antenna, and eye parameters exhibited significant *p*-values above the determined *LC*_50_ concentration, thus indicating toxic effects above this concentration.

#### 3.7.3. Reproduction Rate

**Figure 11:**
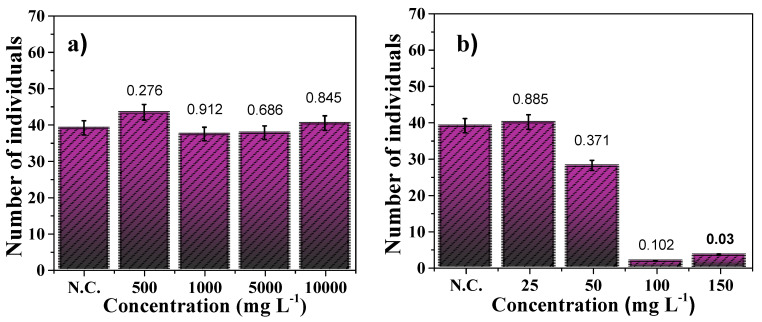
(**a**) Number of individuals born during the Sillar ecotoxicity experiment. (**b**) Number of individuals born during the ecotoxicity experiment with the MS sample. N.C. stands for negative control. Decimal numbers above represent the respective *p*-value compared with N.C. Bold text indicates the data has statistical significance.

Figure 11 shows the mean number of individuals obtained in three replicates for each applied concentration during the ecotoxicity experiment with the Sillar and MS samples, where a significant difference was observed between the two samples. In the case of the Sillar sample (see Figure 11a), no significant differences were found in the number of individuals with respect to the N.C. (see *p*-values above the bars). Nevertheless, in Figure 11b, the MS sample depicted a decrease in the number of individuals as the corresponding concentration increased; with 150 mg L^−1^ being the concentration with statistical significance at which almost no individuals were born. Such a trend was also observed with *D. magna* when exposed to silver NPs [37]. The birth of *D. magna* individuals is a significant result when evaluating the toxic response to NPs. Therefore, while the Sillar sample did not alter the reproduction rate of *D. magna*, the MS sample is highly toxic above its *LC*_50_ value.

### 3.8. Discussion on the Size, Chemical Composition, Dispersion Preparation Method, and Colloidal Stability on the Ecotoxicity Properties

The chemical composition and mean particle size of metal-oxide dispersions have been observed to influence the *LC*_50_ values [17,18,21,34]. For example, reported values of the 24 h-*LC*_50_ for 20–30 nm SiO_2_ and 20 nm Fe_3_O_4_ NPs on *D. magna* were 3704 and 4965.92 mg L^−1^, respectively [38]. In addition, when increasing the exposure time (96 h), the metal-oxide NPs showed increased toxicity to 654. 65 (Fe_3_O_4_) and 1.73 (SiO_2_) mg L^−1^. In another study, 34 nm γ-Fe_2_O_3_ NPs with an exposure time of 72 h on *D. magna* depicted a *LC*_50_ of 50 mg L^−1^ [39], while 6 nm Fe_3_O_4_ NPs showed a 48 h-*LC*_50_ of 23 × 10^−4^ mg L^−1^ [40], which indicates high toxicity for small particle sizes. In our case, the TEM PSD analysis reported a 13 nm γ-Fe_2_O_3_ and the *LC*_50_ was found to be 123.6 mg L^−1^. Therefore, particle size and chemical composition cannot be the only parameters that may influence the *LC*_50_ variability when exposed to *D. magna*. In general, nanosized materials commonly exhibit *LC*_50_ values, in contrast to bulk materials like Sillar.

On the other hand, this changing behavior in the ecotoxicity response can also be associated with the dispersion method (solvents or surfactants, sonication, conventional agitation, and magnetic stirring) and colloidal stability, not reported in the previous studies. It is worth noting that the aging effect during stirring can increase the hydrodynamic size, thereby changing the original NPs physicochemical properties. The sign of the surface potential is of crucial importance, since depending on the positive or negative zeta potential, the composite can attract different ions, hence influencing the *LC*_50_ results. Handy et al. [41] provide an excellent discussion on this topic. Typically, stabilized nano iron oxides depict different zeta potential values that depend on the synthesis and surface functionalization parameters [42]. More importantly, the trend is that the negative potential will be predominant after the p.z.c., and a protonated surface will always occur below this value [25]. However, this did not occur in our MS composite, where the zeta potential was inverted in sign and values of +30 mV were found after pH = 6.4. This last result was atypical and unexpected. Of course, this will be more interesting to study in real effluents containing heavy metals, e.g., where the surface potential can induce bioaccumulation of these pollutants.

### 3.9. Future Perspectives for the Use of the MS Sample

**Scheme 1:**
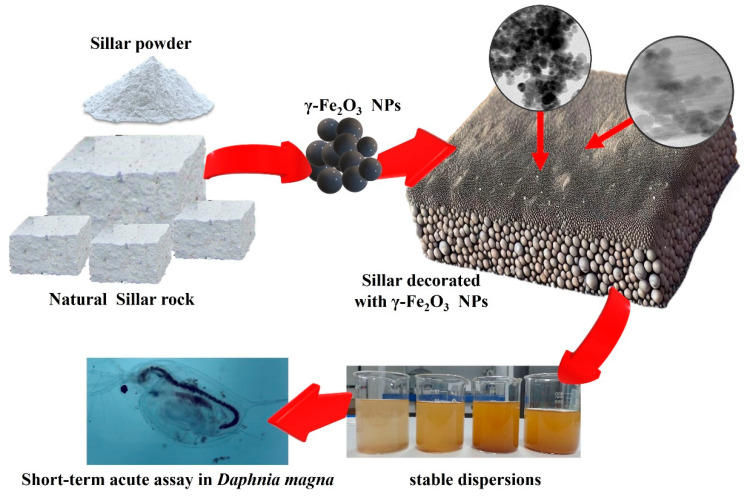
Peruvian pyroclastic dust material decorated with 13 nm $\gamma {-Fe}_{2}O_{3}$ NPs, their stable dispersions, and the 24 h short-term acute assay in *Daphnia magna*.

The present study has established permissible concentrations for treated water effluents. It means that the positively charged MS composite can be applied in the water remediation of anionic pollutants like arsenic, for example. Studies addressing the adsorbent performance of our material will be presented in future work. Finally, Scheme 1 summarizes the prior ecotoxicity protocol to determine the *LC*_50_ for the MS composite.

## 4. Conclusions

For the first time, a novel magnetic composite made of Peruvian Sillar and γ-Fe_2_O_3_ NPs was successfully synthesized. When decorating the surface of Sillar with IONPs, its main crystalline phases were not affected. The particle size distribution (obtained from TEM images) revealed a mean value of 13 nm for γ-Fe_2_O_3_ NPs that was not changed during surface decoration. Magnetization experiments done on pristine Sillar revealed a weak ferromagnetic behavior with low saturation magnetization at 5 K and 300 K. This magnetic behavior can be associated with natural Fe in very low quantities, disposable in Sillar (not quantified by ^57^Fe Mossbauer spectrometry). After decorating the Sillar surface, the γ-Fe_2_O_3_ NPs presented an effective saturation magnetization 50% less than pure γ-Fe_2_O_3_ NPs (60 emu g^−1^). A reasonable explanation can be associated with the volumetric and homogenous distribution of γ-Fe_2_O_3_ NPs into the Sillar framework. At 15 K, the Mössbauer spectrum revealed a trivalent magnetic state only associated with γ-Fe_2_O_3_ NPs. Furthermore, the hyperfine magnetic parameters are as expected for the bulk-like phase, therefore, no atomic interaction or chemical bonding having Fe^3+^ as a participant was observed. The DLS and zeta potential colloidal stability studies determined that the magnetic composite has a p.z.c. of 6.4, which is important for possible applications in aqueous systems, and this value can be contrasted with [18], where the optimum pH range for a similar metal-oxide material was mentioned. Regarding the ecotoxic analysis, it was not possible to find an *LC*_50_ for pure Sillar, under the tested concentrations, while for the MS magnetic composite, its *LC*_50_ was 123.6 mg L^−1^. Above this value, the biomarker showed morphological significant changes in the body, antenna, heart, and eyes. In addition, the reproduction rate was affected at higher concentrations of the magnetic composite. Finally, it is worth mentioning that this work opens the door to new lines of research regarding permissible concentrations for low-cost adsorbent materials in water treatment without causing damage to the *D. magna* ecosystem.

## Data Availability

The original data of this research can be requested any time to the corresponding author’s email address: juan.ramos5@unmsm.edu.pe.

## Supplementary Materials

The following supporting information can be downloaded at: https://www.mdpi.com/article/10.3390/nano14090785/s1, Table S1: Profile broadening parameters used to find the size of the Sillar sample crystallites. Table S2: Corresponding profile broadening parameters used to find the size of the MS sample where the label M means the γ-Fe_2_O_3_ contribution peaks. Table S3: Hyperfine magnetic parameters obtained from the fit of the ^57^Fe Mössbauer spectrum recorded at 300 K for the MS sample. QS indicates the quadrupolar splitting and ε the quadrupolar shifting, W is the Lorentzian line width, R.A.A. is the relative absorption area, CS relates the center shift values, σ is the width of the Gaussian distribution of B_hf_, and B_hf_ is the mean hyperfine magnetic field. Table S4: Hyperfine magnetic parameters obtained from the fit of the ^57^Fe Mössbauer spectrum recorded at 15 K for the MS sample. The quadrupolar shifting (ε) was taken as 0 and the Lorentzian line width was fixed to 0.24, Γ is the line width (mm/s), R.A.A. is the relative absorption area, CS relates the center shift values, and B_hf_ is the mean hyperfine magnetic field.
