# Supplementary material for: Pyroclastic Dust from Arequipa-Peru Decorated with Iron Oxide Nanoparticles and Their Ecotoxicological Properties in Water Flea D. magna
Source: Nanomaterials (Basel). 2024 Apr 30;14(9):785. doi: 10.3390/nano14090785 (PMC11085091; doi:10.3390/nano14090785)
Supplement: Supplementary file 1 [file nanomaterials-14-00785-s001.zip › nanomaterials-2965118-supplementary.pdf]

nanomaterials

MDPI

Article

Supplementary material for Pyroclastic Dust from Arequipa-Peru decorated with iron oxide nanoparticles and their ecotoxicological properties in water flea *D. magna*

Juan A. Ramos-Guivar [1]*, Yacu V. Alca-Ramos[1], Erich V. Manrique-Castillo[1], F. Mendoza-Villa[1], Noemi-Raquel Checca-Huaman[2], Renzo Rueda-Vellasmin[1,3], Edson C. Passamani[3]

Grupo de Investigación de Nanotecnología Aplicada para Biorremediación Ambiental, Energía, Biomedicina y Agricultura (NANOTECH), Facultad de Ciencias Físicas, Universidad Nacional Mayor de San Marcos, Av. Venezuela Cdra 34 S/N, Ciudad Universitaria, Lima 15081, Perú

[2] Centro Brasileiro de Pesquisas Físicas (CBPF), R. Xavier Sigaud, 150, Urca, Rio de Janeiro 22290-180, Brazil

[3] Physics Department, Federal University of Espírito Santo, Vitória 29075-910, Brazil

* Correspondence: juan.ramos5@unmsm.edu.pe

According to Section 2 (Figure 2), the Sillar sample has four identified phases, which are shown in Table S1. To determine the *D* value of each phase, we proceeded using the Equation (1), but before we identified the corresponding contribution of each such phase and calculated the β_{inst} using the relation S1:

$$\beta_{inst} = 0.253 - 0.002 \times (2\theta) + 3.7 \times 10^{-5}(2\theta) \quad \text{(S1)}$$

Table S1. Profile broadening parameters used to find the size of the Sillar sample crystallites.

Phase	$2\theta°$	Cos(θ)	β_{meas}	β_{inst}	β_D	*D* (nm)
Al	13.64	0.992	0.006	0.252	0.004	32
C	21.86	0.981	0.005	0.252	0.003	46
C	23.54	0.978	0.005	0.252	0.002	50
Al	27.73	0.970	0.012	0.252	0.011	12
S	29.81	0.966	0.004	0.252	0.002	67
An	30.16	0.965	0.004	0.252	0.001	144
An	30.70	0.964	0.007	0.252	0.005	26
S	31.29	0.962	0.004	0.252	0.001	102
S	34.76	0.954	0.007	0.252	0.006	24
An	35.95	0.951	0.009	0.252	0.008	19

Table S2 shows the crystallite sizes of the MS sample, for which we identified the relative contributions of each phase, with $\gamma-Fe_2O_3$ overlapping the Sillar phases.

Table S2. Corresponding profile broadening parameters used to find the size of the MS sample where the label M means the $\gamma-Fe_2O_3$ contribution peaks.

Phase	$2\theta°$	Cos(θ)	β_{meas}	β_{inst}	β_D	*D* (nm)
Al	13.46	0.993	0.040	0.252	0.040	4
M	18.32	0.987	0.022	0.252	0.022	7

C	21.87	0.981	0.006	0.252	0.004	32
C	23.58	0.978	0.009	0.252	0.008	17
Al	27.79	0.970	0.011	0.252	0.010	14
M	30.25	0.965	0.016	0.252	0.015	10
M	35.63	0.952	0.014	0.252	0.014	11
M	43.16	0.929	0.016	0.252	0.015	10
M	57.22	0.877	0.012	0.252	0.011	14
M	62.88	0.853	0.014	0.251	0.013	13

Table S3. Hyperfine magnetic parameters obtained from the fit of the ^{57}Fe Mössbauer spectrum recorded at 300 K for the MS sample. QS indicates the quadrupolar splitting and ε the quadrupolar shifting, W is the Lorentzian line width, RA.A. is the relative absorption area, CS relates the center shift values, σ is the width of the Gaussian distribution of B_{hf}, and B_{hf} is the mean hyperfine magnetic field.

	**R.A.A. (%)**	**CS (vs Fe) (mm/s)**	**B_{hf} (T)**	**σ (T)**	**ε or QS (mm/s)**	**W (mm/s)**
**I,A**	20(2)	0.20(1)	47.7(1)	1.2(1)	0.00(2)	0.37(1)
**I,B**	30(2)	0.41(1)	47.7(1)	1.8(1)	0.00(2)	0.37(1)
**II,A**	17(2)	0.22(1)	42.0(1)	4.5(2)	0.00(2)	0.37(1)
**II,B**	28(2)	0.43(1)	42.0(1)	4.5(2)	0.00(2)	0.37(1)
**doublet**	5(2)	0.32(4)	-	-	0.78(7)	0.37(4)

Table S4. Hyperfine magnetic parameters obtained from the fit of the ^{57}Fe Mössbauer spectrum recorded at 15 K for the MS sample. The quadrupolar shifting (ε) was taken as 0 and the Lorentzian line width was fixed to 0.24, Γ is the line width (mm/s), R.A.A. is the relative absorption area, CS relates the center shift values, and B_{hf} is the mean hyperfine magnetic field.

	**R.A.A. (%)**	**CS (vs Fe) (mm/s)**	**B_{hf} (T)**	**Γ (mm/s)**
**A**	37(2)	0.39 (2)	51.0 (2)	0.34(1)
**B**	63(2)	0.47 (2)	52.8 (2)	0.35(1)
